# Is patients’ rurality associated with in-hospital sepsis death in US hospitals?

**DOI:** 10.3389/fpubh.2023.1169209

**Published:** 2023-06-13

**Authors:** Jongwha Chang, Mar Medina, Sun Jung Kim

**Affiliations:** ^1^Department of Pharmaceutical Sciences, Irma Lerma Rangel School of Pharmacy, Texas A&M University, College Station, TX, United States; ^2^School of Pharmacy, University of Texas at El Paso, El Paso, TX, United States; ^3^Department of Health Administration and Management, College of Medical Science, Soonchunhyang University, Asan, Republic of Korea; ^4^Center for Healthcare Management Science, Soonchunhyang University, Asan, Republic of Korea; ^5^Department of Software Convergence, Soonchunhyang University, Asan, Republic of Korea

**Keywords:** rurality, sepsis, NIS sample, in-hospital death, health disparity

## Abstract

**Background:**

The focus of this study was to explore the association of patients’ rurality and other patient and hospital-related factors with in-hospital sepsis mortality to identify possible health disparities across United States hospitals.

**Methods:**

The National Inpatient Sample was used to identify nationwide sepsis patients (*n* = 1,977,537, weighted *n* = 9,887,682) from 2016 to 2019. We used multivariate survey logistic regression models to identify predictors for how patients’ rurality is associated with in-hospital death.

**Findings:**

During the study periods, in-hospital death rates among sepsis inpatients continuously decreased (11.3% in 2016 to 9.9% in 2019) for all rurality levels. Rao-Schott Chi-Square tests demonstrated that certain patient and hospital factors had varied in-hospital death rates. Multivariate survey logistic regressions suggested that rural areas, minorities, females, older adults, low-income, and uninsured patients have higher odds of in-hospital mortality. Further, specific census divisions like New England, Middle Atlantic, and East North Central had greater in-hospital sepsis death odds.

**Conclusion:**

Rurality was associated with increased in-hospital sepsis death across multiple patient populations and locations. Further, rurality in New England, Middle Atlantic, and East North Central locations is exceptionally high odds. In addition, minority races in rural areas also have an increased odds of in-hospital death. Therefore, rural healthcare requires a more significant influx of resources and should also include assessing patient-related factors.

## Background

Sepsis, or septicemia, as defined by the Center for Disease Control (CDC), is caused by the body’s large-scale reaction to an infection resulting in a medical emergency that affects 1.7 million Americans annually (1, 2). Many infections, like respiratory, urinary, or skin infections, by bacteria, viruses, or fungi, can devolve into sepsis, requiring rapid diagnosis and treatment to prevent organ failure and death (1). At-risk populations include patients over 65, immunocompromised, recently hospitalized, or severely ill (1). The CDC estimates that in 1 in 3 hospital deaths, that patient likely had sepsis during hospitalization and that 87% of sepsis cases occurred outside the hospital (1). There is also growing concern for antibiotic-resistant organisms, with dangerous implications for fluoroquinolone-resistant *E. coli* (3). Because sepsis is widespread, it is vital to understand risk factors to target prevention efforts. For instance, there may be regional associations for some sepsis-associated conditions like sepsis associated-in-hospital cardiac arrest (SA-IHCA), with the South having increased SA-IHCA incidence rates and the West with the highest mortality rates (4). As some described, the “Sepsis Belt” includes impoverished communities in Alabama, Arkansas, Georgia, Louisiana, Mississippi, North Carolina, South Carolina, and Tennessee (5).

Prevention and identification are critical across populations because of sepsis’s threat to human life. Sepsis continues to be a leading cause of hospitalization, death, cost, and morbidity (3, 6, 7). Sepsis can lead to rehospitalization and decreased quality of life (6). Older adults and nursing home residents are also at an increased risk for severe disease, extended hospital stays, ICU rates, and in-hospital mortality (8, 9). Compared to patients with comorbidities, if sepsis is not diagnosed early (10), even previously healthy adults may experience poor outcomes.

Previous literature has described differences in health outcomes for sepsis patients based on race and ethnicity, finding higher mortality rates for all minority racial/ethnic patients than white patients (11). Race as an independent factor is insufficient to explain the health disparities in sepsis outcomes but demonstrates concerning trends (12). Past literature contends that because sepsis treatment is standardized, differences in outcomes indicate health disparities (13). Likely, increased comorbidities, lower insurance rates, and more significant limitations in their built environment for racial and ethnic minorities could explain their worse sepsis outcomes (13). Multiple studies found that African Americans have some of the highest sepsis mortality rates (12, 13); however, Engoren and Arslanian-Engoren had contradictory results in a single-center study that found African Americans have some of the lowest mortality rates (14). Racial disparities have been well described in past research (13, 15) and require further investigation to illuminate the extent of and prevalence of it in sepsis patients.

Another type of disparity impacting sepsis outcomes is the urban–rural difference. The CDC recognizes the urban–rural difference with health effects being monitored and compared between patients in various levels of urbanization and rurality (16). In 2018, the United States Department of Agriculture showed that rural areas tend to be less diverse than urban areas, with 78.2% of the population being White compared to 57.3% in urban areas, almost a 20% difference (17). The urban–rural difference in care can also be studied by race and ethnicity to compare how the two factors may exacerbate one another. One study explains that the black-and-white difference is further compounded by the rural and urban mortality disparity and fueled by discrepancies in sociodemographic characteristics, yet public health efforts have contradictory effects (18). For example, rural public health efforts may reduce black mortality rates yet increase white mortality rates, possibly due to differences in poverty levels and access; hence, blanket measures cannot account for targeted needs (18).

The difference in health outcomes is further displayed when comparing uninsured rural and urban patients, with rural uninsured patients having higher in-hospital mortality rates (19). Limited access to specialists, distance to providers, and financial barriers are common rural issues (20, 21). The limitation of resources in rural areas was tested during the COVID-19 pandemic, in which calls for greater resource allocation to non-urban areas increased, and strained healthcare systems began to take on water (22, 23). Multiple studies have demonstrated unique features in rural hospitals and patients regarding how rurality affects sepsis rates and outcomes. For example, in one rural hospital with a health professional shortage, indwelling medical devices, commonly urinary catheters, inserted in patients with more prolonged hospitalizations increase the risk of sepsis more than if inserted into patients with shorter stays (24). These results are repeated in a study by Ahiawodzi et al., which also found that patients with government health insurance (Medicare, Medicaid, or both) in a rural hospital have an increased risk of sepsis diagnosis (25).

Medicare enacted a sepsis quality improvement policy, but safety net hospitals (hospitals that mainly treat uninsured, Medicaid, or low-income patients) (26) have been underperforming in such standards (27). In one study, safety net hospitals tended to be non-affiliated with healthcare systems, be teaching hospitals and owned publicly or by the government, and have demonstrated a lower quality of sepsis care compared to hospitals treating patients with higher socioeconomic status (27). Further, uninsured patients with community-acquired sepsis tend to be admitted at later stages of the disease; therefore, insurance status could be an essential hurdle to care (28). Rural areas have made some progress in lowering the uninsured rate in their population with the Affordable Care Act, but uninsured rates in these regions are still higher than in urban locations (29).

Despite the high prevalence of sepsis in the United States and the documented limitations of rurality on public health and sepsis outcomes, more research is required to characterize the patient and hospital factors associated with rurality and in-hospital death from sepsis. Such information can direct resources to specific populations and illuminate any racial or sociodemographic disparities exacerbated in rural communities. Therefore, this study seeks to identify patient and hospital factors associated with sepsis outcomes, such as race, location, and rurality levels. Specifically, we will focus on hospitalized patients using National Inpatient Sample data to reflect on relevant health disparities demonstrated by populations at increased odds of death from sepsis. Discussing health inequalities is essential for improving the health of Americans and understanding the challenges communities and hospitals face in promoting proper care.

## Materials and method

### Data collection

**Figure 1:**
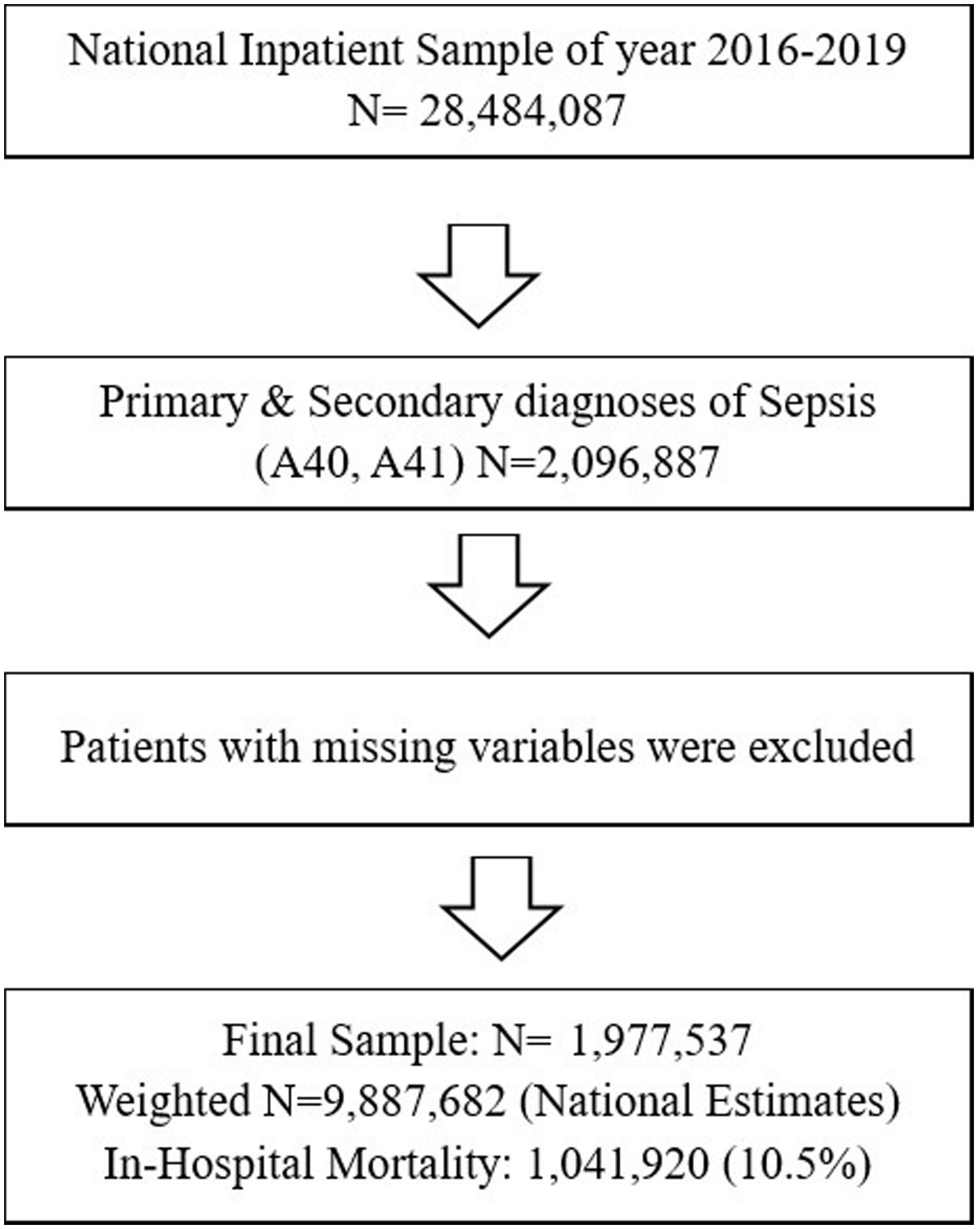
Flow chart of sample selection.

This study used the latest 2016–2019 National Inpatient Sample Database (NIS). It is a serial, cross-sectional, retrospective data set, a product of the Healthcare Cost and Utilization Project (HCUP), sponsored by the Agency for Healthcare Research and Quality (AHRQ). The NIS data set is the largest publicly available all-payer inpatient database in the United States and includes data from nearly 8 million hospital inpatient stays. The NIS represents a 20% sample of all nonfederal, short-term hospitals from 44 states in the United States. We used the International Classification of Diseases Tenth Revision (ICD-10-CM/PCS) codes for sepsis (ICD-10-CM/PCS code A40, A41) to identify patients with primary and secondary diagnoses of sepsis (*n* = 2,096,887) from all 2016 to 2019 NIS samples (*N* = 28,484,087). Thus, we obtained a population-based estimate for nationwide sepsis cases. Afterward, we excluded patients with missing variables, resulting in our final sepsis patient sample (*n* = 1,977,537, weighted *n* = 9,887,682). This process is demonstrated in Figure 1.

### Variables

To investigate the association between rurality and in-hospital sepsis mortality, we set the “Died during hospitalization” variable as the primary outcome and rurality as the primary predictor. Rurality is defined by the patient’s county of residence and the six-category urban–rural classification scheme for US counties developed by the National Center for Health Statistics and reported in the NIS database (16). The six categories are (1) central metro counties of at least 1 million people, (2) large fringe metro counties, (3) medium fringe metro counties, (4) small fringe metro counties, (5) micropolitan counties, and (6) noncore counties. Like previous literature, we collapsed these categories into three levels: Urban (1), suburban (2–4), and rural (5 and 6) (16, 17). We also adjusted various patient and hospital confounders. Patient characteristics included age, race, annual median household income, primary payer (Medicare, Medicaid, Self-Pay/No Charge, Other, and Private insurance), and illness severity (All Patient Refined DRGs: the severity of illness subclass and the risk of mortality subclass within each base APR-DRG.). Hospital characteristics include bed size (For teaching hospitals, <250(Small), 250 ~ 450(Medium), and 450 > (Large). For non-teaching hospitals, <100(Small), 100 ~ 200(Medium), and 200 > (Large)), ownership, teaching status, and region of the hospital.

### Statistical analysis

Sampling weights were applied to all statistical analyses to represent nationwide sepsis patients. We first studied the patient and hospital characteristics of the final dataset by in-hospital mortality, presented as weighted frequency (percentage) and means (SD) in Table 1. Next, Rao-Schott Chi-Square tests for categorical variables was used to investigate group differences. Then, a multivariate survey logistic regression analysis was used to explore rurality with in-hospital death, adjusting for patient and hospital characteristics (Table 2). We also ran the model using replaced census division variable (Table 3) and analyzed sub-groups by race and census division (Table 4) while adjusting all other variables. Sub-group analysis equates to stratified analysis, which means we separated the sample into various subgroups presented in Table 4 and ran the model using only the separated sample population. All statistical tests were two-sided, and statistical significance was determined at a value of *p* < 0.05. All studies used SAS statistical software (version 9.4; SAS Institute Inc., Cary, NC, United States).

**Table 1:** General characteristics of sample.

Variables	Total		In-hospital death						*p*
	*N*/Mean	%	No			Yes			
			*N*	Row %	Column %	*N*	Row %	Column %	
*N*	1,977,537		1,769,153	89.5		208,384	10.5		
Weighted *N* [national estimates]	9,887,682		8,845,763	89.5		1,041,920	10.5		
Rurality: patients’ residence									
Urban	3,002,640	30.4	2,673,200	89.0	30.2	329,440	11.0	31.6	<0.0001
Sub-urban	5,325,909	53.9	4,782,399	89.8	54.1	543,510	10.2	52.2	
Rural	1,559,133	15.8	1,390,164	89.2	15.7	168,970	10.8	16.2	
Race									
White	6,781,393	68.6	6,062,093	89.4	68.5	719,300	10.6	69.0	<0.0001
Black	1,377,975	13.9	1,226,485	89.0	13.9	151,490	11.0	14.5	
Hispanic	1,082,000	10.9	985,820	91.1	11.1	96,180	8.9	9.2	
Asian or pacific islander	302,335	3.1	267,115	88.4	3.0	35,220	11.6	3.4	
Other	343,980	3.5	304,250	88.4	3.4	39,730	11.6	3.8	
Age									
~ 44	1,481,520	15.0	1,421,315	95.9	16.1	60,205	4.1	5.8	<0.0001
45 ~ 64	2,939,799	29.7	2,675,684	91.0	30.2	264,115	9.0	25.3	
65 ~ 74	2,209,265	22.3	1,955,880	88.5	22.1	253,385	11.5	24.3	
75~	3,257,099	32.9	2,792,884	85.7	31.6	464,215	14.3	44.6	
Sex									
Male	4,996,799	50.5	4,456,524	89.2	50.4	540,275	10.8	51.9	<0.0001
Female	4,890,884	49.5	4,389,239	89.7	49.6	501,645	10.3	48.1	
Median household income									
0–25th percentile	3,104,509	31.4	2,773,984	89.4	31.4	330,525	10.6	31.7	<0.0001
26th–50th percentile	2,585,179	26.1	2,319,064	89.7	26.2	266,115	10.3	25.5	
51st–75th percentile	2,335,374	23.6	2,095,479	89.7	23.7	239,895	10.3	23.0	
76th–100th percentile	1,862,620	18.8	1,657,235	89.0	18.7	205,385	11.0	19.7	
Primary payer									
Medicare	6,027,933	61.0	5,311,084	88.1	60.0	716,850	11.9	68.8	<0.0001
Medicaid	1,465,655	14.8	1,353,710	92.4	15.3	111,945	7.6	10.7	
Private insurance	1,779,125	18.0	1,628,310	91.5	18.4	150,815	8.5	14.5	
Self-pay	362,905	3.7	335,840	92.5	3.8	27,065	7.5	2.6	
No charge	31,175	0.3	29,720	95.3	0.3	1,455	4.7	0.1	
Other	220,890	2.2	187,100	84.7	2.1	33,790	15.3	3.2	
Severity of illness									
No/minor comorbidity or complications	278,545	2.8	277,315	99.6	3.1	1,230	0.4	0.1	<0.0001
Moderate comorbidity or complications	1,717,244	17.4	1,705,289	99.3	19.3	11,955	0.7	1.1	
Major comorbidity or complications	3,586,154	36.3	3,474,284	96.9	39.3	111,870	3.1	10.7	
Extreme comorbidity or complications	4,305,740	43.5	3,388,875	78.7	38.3	916,865	21.3	88.0	
Bed-size of hospital									
Small	2,029,737	20.5	1,855,262	91.4	21.0	174,475	8.6	16.7	<0.0001
Medium	2,956,513	29.9	2,651,753	89.7	30.0	304,760	10.3	29.2	
Large	4,901,432	49.6	4,338,747	88.5	49.0	562,685	11.5	54.0	
Ownership of hospital									
Government, nonfederal	1,029,467	10.4	905,932	88.0	10.2	123,535	12.0	11.9	<0.0001
Private, non-profit	7,358,421	74.4	6,585,936	89.5	74.5	772,485	10.5	74.1	
Private, invest-own	1,499,795	15.2	1,353,895	90.3	15.3	145,900	9.7	14.0	
Teaching status of the hospital									
Teaching	6,650,959	67.3	5,899,514	88.7	66.7	751,445	11.3	72.1	<0.0001
Non-teaching	3,236,724	32.7	2,946,249	91.0	33.3	290,475	9.0	27.9	
Region of hospital									
Northeast	1,703,895	17.2	1,498,535	87.9	16.9	205,360	12.1	19.7	<0.0001
Midwest	1,996,355	20.2	1,803,260	90.3	20.4	193,095	9.7	18.5	
South	3,951,051	40.0	3,532,181	89.4	39.9	418,870	10.6	40.2	
West	2,236,382	22.6	2,011,787	90.0	22.7	224,595	10.0	21.6	

**Table 2:** Results of multivariate survey logistic regression model: factors associated with in-hospital death.

Variables	Odds ratio	95% CLs	
Rurality: patients’ residence			
Urban	Ref.		
Sub-urban	0.989	0.978	1.001
Rural	1.119	1.100	1.138
Race			
White	Ref.		
Black	1.048	1.032	1.063
Hispanic	0.999	0.981	1.016
Asian or pacific islander	1.095	1.064	1.125
Other	1.141	1.112	1.171
Age			
~ 44	Ref.		
45 ~ 64	1.935	1.894	1.976
65 ~ 74	2.684	2.621	2.748
75~	3.720	3.634	3.808
Sex			
Male	0.954	0.945	0.963
Female	Ref.		
Median household income			
0–25th percentile	Ref.		
26th–50th percentile	0.971	0.958	0.984
51st–75th percentile	0.953	0.940	0.966
76th–100th percentile	0.972	0.958	0.987
Primary payer			
Medicare	0.801	0.789	0.814
Medicaid	0.942	0.924	0.961
Private insurance	Ref.		
Self-pay	1.218	1.179	1.258
No charge	0.792	0.701	0.895
Other	1.551	1.503	1.602
Severity of illness			
No/minor comorbidity or complications	Ref.		
Moderate comorbidity or complications	1.258	1.104	1.435
Major comorbidity or complications	5.206	4.590	5.905
Extreme comorbidity or complications	44.095	38.902	49.982
Bed-size of hospital			
Small	Ref.		
Medium	1.137	1.120	1.154
Large	1.221	1.204	1.238
Ownership of hospital			
Government, nonfederal	Ref.		
Private, non-profit	0.852	0.839	0.866
Private, invest-own	0.846	0.830	0.863
Teaching status of the hospital			
Teaching	1.188	1.174	1.201
Non-teaching	Ref.		
Region of hospital			
Northeast	1.300	1.279	1.321
Midwest	Ref.		
South	1.141	1.126	1.158
West	1.096	1.079	1.114
Year			
2016	Ref.		
2017	0.931	0.918	0.944
2018	0.781	0.770	0.791
2019	0.860	0.848	0.872

**Table 3:** Results of multivariate survey logistic regression model using census division of hospital variable.

Variables	Odds ratio	95% CLs	
Rurality: patients’ residence			
Urban	Ref.		
Sub-urban	0.997	0.986	1.009
Rural	1.120	1.101	1.139
Race			
White	Ref.		
Black	1.046	1.030	1.061
Hispanic	0.999	0.981	1.017
Asian or pacific islander	1.064	1.034	1.094
Other	1.148	1.118	1.178
Census division of the hospital			
New England	1.131	1.102	1.160
Middle Atlantic	1.184	1.163	1.204
East North Central	0.900	0.885	0.916
West North Central	0.903	0.882	0.925
South Atlantic	Ref.		
East South Central	1.132	1.109	1.156
West South Central	1.004	0.986	1.023
Mountain	0.830	0.811	0.850
Pacific	1.057	1.039	1.076

*All other variables were adjusted.

**Table 4:** Results of multivariate survey logistic regression model: sub-groups analysis by race and region.

Variables	Sub-urban			Rural		
	Odds ratio	95% CLs		Odds ratio	95% CLs	
Race						
White	0.989	0.974	1.003	1.113	1.091	1.136
Black	1.006	0.978	1.035	1.176	1.120	1.236
Hispanic	0.959	0.927	0.992	1.027	0.956	1.104
Asian or pacific islander	0.953	0.899	1.011	0.997	0.838	1.186
Other	0.988	0.934	1.046	1.167	1.074	1.268
Census division of the hospital						
New England	1.050	0.986	1.120	1.107	1.003	1.222
Middle Atlantic	0.883	0.857	0.911	1.034	0.974	1.097
East North Central	1.056	1.023	1.091	1.182	1.129	1.236
West North Central	1.020	0.955	1.090	1.161	1.074	1.254
South Atlantic	1.001	0.972	1.030	1.095	1.051	1.140
East South Central	1.060	1.006	1.118	1.197	1.128	1.271
West South Central	1.012	0.979	1.047	1.158	1.107	1.210
Mountain	1.039	0.992	1.089	1.315	1.229	1.406
Pacific	1.013	0.987	1.041	1.032	0.972	1.096

*Reference is Urban. All other variables were adjusted.

## Results

### Patient/hospital characteristics and descriptive statistics

A total of 1,977,537 sepsis patients were identified in the 2016 to 2019 NIS data (weighted *n* = 9,887,682, Table 1). From them, 208,384 (weighted *n* = 1,041,920, 10.5%) died during hospitalization. General patient and hospital characteristics are displayed in Table 1.

Table 5 holds the temporal trends of nationwide sepsis in-hospital death from 2016 to 2019. For all rurality levels across the study period, there was a decreasing in-hospital mortality rate for national sepsis patients; there was an 11.3% death in 2016 and a 9.9% death in 2019.

**Table 5:** Temporal trend of in-hospital death of sepsis patients.

	2016		2017		2018		2019	
*N*	431,499		486,420		521,822		537,796	
Weighted *N* [national estimates]	2,157,494		2,432,099		2,609,110		2,688,980	
Sepsis inpatients by rurality								
Urban	631,870	29.3%	745,695	30.7%	806,770	30.9%	818,305	30.4%
Sub-urban	1,170,949	54.3%	1,304,400	53.6%	1,396,361	53.5%	1,454,200	54.1%
Rural	354,675	16.4%	382,004	15.7%	405,979	15.6%	416,475	15.5%
Death during the hospitalization								
Total	243,160	11.3%	263,225	10.8%	270,045	10.4%	265,490	9.9%
Urban	75,775	12.0%	83,640	11.2%	86,675	10.7%	83,350	10.2%
Sub-urban	126,925	10.8%	137,935	10.6%	139,505	10.0%	139,145	9.6%
Rural	40,460	11.4%	41,650	10.9%	43,865	10.8%	42,995	10.3%

### Association between rurality and in-hospital death

Table 2 displays the multivariate survey logistic regression model on the association between patient rurality and in-hospital death. After controlling for all other variables, rural patients are associated with greater odds of in-hospital death than urban patients (OR = 1.119, 95% CI = 1.100, 1.138). In addition, older adults, females, low-income, uninsured patients, or those with severe comorbidities/complications are at increased odds of in-hospital death. Further, minority races and ethnicities demonstrate increased odds of in-hospital sepsis death compared to white patients. Finally, teaching hospitals or the Northeast, South, and West are at greater odds of in-hospital death. This result is repeated for large and government-owned hospitals.

Table 3 has the multivariate survey logistic regression using census divisions for the region variable. Rural patients in New England, Middle Atlantic, East South Central, and the Pacific had some of the highest odds of in-hospital mortality. Table 4 holds the stratified sub-group analysis for race and census division and shows that rural residents are at greater odds for in-hospital mortality than urban residents across all categories.

## Discussion

Analyzing in-hospital sepsis mortality can identify populations for worse sepsis outcomes and promote targeted prevention, identification, and resource allocation. We identified various patient factors associated with in-hospital sepsis death and explored rurality’s effect on mortality rates. Similar to previous literature, sepsis outcomes are worse for uninsured patients (25, 28). In our research, self-pay and other-payer options were associated with increased odds of in-hospital sepsis death. Further, Medicaid, though slightly less likely, had the highest odds of sepsis mortality of the government insurance options like the studies by Ahiawodzi et al. (25). Although uninsured rates have decreased in rural areas (29), our results show that further work is needed to reduce uninsured and improve patient outcomes on government insurance. Low-income patients also demonstrated slightly higher odds of in-hospital sepsis mortality than other incomes; thus, when combined with the risks associated with uninsured and government insurance, patients may face more complex healthcare barriers. Medium and large hospitals were also associated with higher sepsis death, elucidating another disparity. Past literature describes how large rural hospitals are more likely to be involved in mergers (30) and that patients report lower satisfaction in larger hospitals (31). Urban hospitals may contribute to this finding as previous literature has also found better sepsis performance in smaller hospitals (27, 32). Therefore, more research is required to explore the quality of care in large rural and urban hospitals compared to small hospitals. Non-government hospitals also had statistically significantly lower odds of in-hospital sepsis death than government hospitals. This finding is similar to previous literature, which described how smaller for-profit hospitals tend to have better SEP-1 performance than other hospitals (a measure of sepsis performance), and government hospitals had the lowest SEP-1 performance (27, 32).

Compared to urban patients, sub-urban and rural patients have higher odds of in-hospital sepsis death, though only rural areas were statistically significant. These results are repeated even when using the more specific census division variable. Our results are like previous research, which often finds that rural patients have worse outcomes for multiple health issues (16–19). A past study describes those rural sepsis patients present to hospitals at late stages, which aligns with our results (28). Rural patients may suffer from increased complications or comorbidities (33–35) and not present for medical care at more manageable points in their disease for monetary or distance-to-treatment reasons (20, 21). Indeed, we have found that the odds of in-hospital death increase exponentially with more significant comorbidities or complications. In addition, previous research found that uninsured rural patients have worse mortality rates (19, 28), an outcome reflected in our study with worse odds for mortality for uninsured and rural patients. Therefore, providing hospitals with more resources is necessary but insufficient to curb sepsis because there are multiple exacerbating patient factors; patients require increased early access to care and better primary care management. Rural regions need multiple assistance points to improve in-hospital sepsis mortality.

Looking at location, we found that all regions compared to the Midwest had higher in-hospital sepsis mortality, with the highest odds in the Northeast. Going into more specific Census Divisions, the highest odds were in the Middle Atlantic, which is in the Northeast, and the lowest was in the Mountain region in the West. However, the sub-group analysis by race and region demonstrates slightly different and more specified results. For all Census Divisions, rural areas had higher odds ratios for sepsis in-hospital mortality than sub-urban areas in those regions. The highest odds ratios were in rural New England, Mountain, and East North Central. These areas have a sharp mix of urban cities to more rural locations (16). For example, most of the Mountain region is considered micropolitan or noncore cities, which we classified as rural areas. New England has a sparse amount of large central metropolitan counties, but the majority are either sub-urban or rural; Maine, for instance, is almost entirely rural. East North Central is more divided into small counties and has a broader mix of urban, suburban, and rural areas; still, some states, like Wisconsin and northern Michigan, are primarily rural.

For comparison, the Middle and South Atlantic had two of the lowest odds ratios for rural regions in our sub-groups analysis. Those areas also have a more significant conglomeration of major urban cities surrounded by suburban areas. While there are still rural areas in the Middle and South Atlantic, they are more dispersed by urbanized regions. Our results are significant because they demonstrate how concentrated areas of rural patients have worse sepsis outcomes. Furthermore, across all divisions, rurality was associated with higher odds of in-hospital mortality than in suburban areas; thus, patients who live in rural areas are not receiving proper care. The reasons for this disparity are complex, ranging from physical distance to finances, provider shortages, comorbidities, and insurance status (19–21, 28, 33–35). However, our research demonstrates that more research and resources must focus on patient factors rather than only hospital performance to improve this health inequality.

Further demonstrating the importance of patient factors, race, and rurality were also associated with disparities in in-hospital death. Again, all races in rural settings had worse outcomes than their suburban counterparts. In the suburbs, the races had comparable odds for in-hospital sepsis mortality rates except for Black patients. Only suburban Black patients were associated with higher odds for in-sepsis mortality (though not statistically significant). Potential reasoning for this trend comes from the Shao et al. study, which found that Black and Hispanic neighborhoods had lower accessibility to healthcare compared to white communities in Chicago (36). Although this study found that most Black communities were in the city’s center, and most healthcare was found around the city’s outskirts, it demonstrates that different racial and ethnic neighborhoods have separate access to care due to economic and transit factors (36). Further research is required to understand Black suburban patients’ barriers to accessing care compared to their White or rural counterparts. The odds of sepsis in-hospital mortality rates are increased for all races in rural areas.

Our results demonstrate that rurality, across all races and ethnicities, is associated with a greater risk for in-hospital sepsis death. Each race and ethnicity have higher odds of in-hospital sepsis death in rural locations than in urban areas (Table 4). Rurality seems to have an additive effect on all races and ethnicities and increases their odds of in-hospital death; the rural odds ratios for White and Black patients were all statistically significant, while the sub-urban odds were not. The inverse is true for Hispanic patients. If there were equal treatment between races and ethnicities, we would expect the association between races and in-hospital sepsis mortality in rural areas to be lower for minority patients considering the lack of diversity in rural populations (17). However, despite less diversity in rural areas (17), Black patients had higher odds of in-hospital sepsis mortality compared to White patients. These results may be significant because the risk is practically identical, but the populations are not. While White patients’ high odds ratio may be explained by being the most considerable portion of the sample in rural areas, the same cannot be said of Black patients and may indicate a relevant healthdisparity.

Furthermore, our results in Tables 2 and 3 indicate that all other minority races and ethnicities are associated with higher in-hospital sepsis mortality than white patients. Black and Asian or Pacific Islander patients had statistically significant increased odds of in-hospital death compared to White patients, and Hispanic patients also had increased odds, but the finding was not statistically significant. Previous research has identified racial health inequalities (11–13, 15, 17, 18, 36), and our findings indicate the need for further research to explore the association between race and in-hospital mortality by rurality. Racial health inequalities are well-engrained by historical sociodemographic segregation, economic and educational disadvantages, and unequal power balance (37–39). Thus, achieving health equality is an intricate task involving communities and addressing patient-specific cultural needs or concerns.

This study has identified the connection between rurality and other patient and hospital-related factors with in-hospital sepsis death. Still, there are some limitations to our research. First, the National Inpatient Sample dataset uses ICD-10 codes for sepsis, limiting patient selection. ICD-10 codes may miss cases with limited information or miscode patients based on symptoms alone (40). Second, this dataset does not include clinical information on disease severity, limiting real-life interpretation and weakening the study results. For example, clinical factors that could influence sepsis mortality include organism or infection type. In addition, this dataset does not include detailed ethnicity information, limiting our analysis to the five races and ethnicities used. Results also show that Hispanic patients had increased odds of in-hospital death, but the finding was not significant and thus requires further study to elucidate its possible association with sepsis mortality. Additional analysis using characteristics of hospital transfers also needs to be conducted. The dataset also cannot account for rural patients who are transferred or seek treatment in urban or sub-urban hospitals, which could impact results. Because of its large sample sizes, the *p*-value for the Rao-Schott Chi-Square test might be affected. Finally, the dataset does not contain patient and physician perspectives on sepsis care quality between inpatient and outpatient locations. Despite these limitations, our study includes essential information on rural health inequalities by location, race, and other patient and hospital-related factors.

## Conclusion

This study sought to discern how rurality affected sepsis in-hospital death and explore possible patient and hospital factors that also increase mortality odds. Rurality was repeatedly shown to increase the odds of in-hospital sepsis death and was identified in specific census divisions like New England, Middle Atlantic, and East North Central. Rural locations across the US are lagging in appropriate sepsis care, but the identified regions in this study showed higher in-hospital mortality odds. Therefore, hospital managers and policymakers in these regions should focus on their patient populations to improve health outcomes and hospital performance. Other patient factors that may increase sepsis mortality odds include uninsured/self-pay, low income, higher comorbidities or complexity, and minority race or ethnicity. Our research has highlighted differences in sepsis outcomes for rural patients that warrant further study and improvement in patient-specific care. For example, rural minority ethnicity patients may be at increased odds for sepsis mortality. They may require improved rural healthcare and culturally sensitive care assessments to catch them at earlier presentations. Without targeted patient-centered interventions, blanket actions and hospital-level changes will not be sufficient to curtail the rural healthcare crises and allow racial health inequalities to continue.

## Data availability statement

The original contributions presented in the study are included in the article/supplementary material, further inquiries can be directed to the corresponding author.

## Ethics statement

The studies involving human participants were reviewed and approved by this study was approved for waiver from the Institutional Review Board, Soonchunhyang University (202203-SB-027). Written informed consent for participation was not required for this study in accordance with the national legislation and the institutional requirements.

## Author contributions

JC and SK led the design and conception of the study, performed the data analysis, and edited the manuscript. MM contributed to writing the first draft and revising the manuscript. SK is the guarantor of this work and, as such, has full access to all the data in the study and takes responsibility for the integrity of the data and accuracy of the data analysis. All authors contributed to the article and approved the submitted version.

## Funding

This paper was supported by Soonchunhyang University Research Fund, the Basic Science Research Program through the National Research Foundation of Korea (NRF) funded by the Ministry of Education (2022R1F1A1063423), and BK21 FOUR (Fostering Outstanding Universities for Research), No.:5199990914048, Korean Ministry of Education. The funding sources did not have study design and data interpretation interventions.

## Conflict of interest

The authors declare that the research was conducted in the absence of any commercial or financial relationships that could be construed as a potential conflict of interest.

## Publisher’s note

All claims expressed in this article are solely those of the authors and do not necessarily represent those of their affiliated organizations, or those of the publisher, the editors and the reviewers. Any product that may be evaluated in this article, or claim that may be made by its manufacturer, is not guaranteed or endorsed by the publisher.
